# Value of gadoxetic acid-enhanced MRI for microvascular invasion of small hepatocellular carcinoma: a retrospective study

**DOI:** 10.1186/s12880-021-00572-w

**Published:** 2021-03-05

**Authors:** Meng Zhou, Dan Shan, Chunhui Zhang, Jianhua Nie, Guangyu Wang, Yanqiao Zhang, Yang Zhou, Tongsen Zheng

**Affiliations:** 1 Department of Gastrointestinal Medical Oncology, Harbin Medical University Cancer Hospital, No.150 Haping Road, Nangang District, Harbin, 150081 Heilongjiang People’s Republic of China; 2 Department of Radiology, Harbin Medical University Cancer Hospital, No. 150 Haping Road, Nangang District, Harbin, 150001 Heilongjiang People’s Republic of China; 3 Department of Phase 1 Trials Center, Harbin Medical University Cancer Hospital, Harbin, Heilongjiang People’s Republic of China; 4 Heilongjiang Cancer Institute, Harbin, Heilongjiang People’s Republic of China

**Keywords:** Small hepatocellular carcinoma, Spicule sign, Rim enhancement, Capsule enhancement

## Abstract

**Background:**

The objective of this study was to analyze the accuracy of gadolinium–ethoxybenzyl–diethylenetriamine penta–acetic acid enhanced magnetic resonance imaging (Gd–EOB–DTPA–MRI) for predicting microvascular invasion (MVI) in patients with small hepatocellular carcinoma (sHCC) preoperatively.

**Methods:**

A total of 60 sHCC patients performed with preoperative Gd–EOB–DTPA–MRI in the Harbin Medical University Cancer Hospital from October 2018 to October 2019 were involved in the study. Univariate and multivariate analyses were performed by chi–square test and logistic regression analysis. The sensitivity, specificity, accuracy, positive predictive value, and negative predictive value of Gd–EOB–DTPA–MRI were performed by receiver operating characteristic (ROC) curves.

**Results:**

Univariate analysis indicated that alanine aminotransferase (≥ 39.00U/L), poorly differentiated pathology, and imaging features including grim enhancement, capsule enhancement, arterial halo sign and hepatobiliary features (tumor highly uptake, halo sign, spicule sign and brush sign) were associated with the occurrence of MVI (*p* < 0.05). Multivariate analysis revealed that rim enhancement and hepatobiliary spicule sign were independent predictors of MVI (*p* < 0.05). The area under the ROC curve was 0.917 (95% confidence interval 0.838–0.996), and the sensitivity was 94.74%.

**Conclusions:**

The morphologies of hepatobiliary phase imaging, especially the spicule sign, showed high accuracy in diagnosing MVI of sHCC. Rim enhancement played a significant role in diagnosing MVI of sHCC.

## Background

Hepatocellular carcinoma (HCC) accounts for the majority of malignant primary hepatic tumors [1]. It ranks sixth in terms of incidence and is the fourth most common cause of cancer mortality worldwide [2]. Small hepatocellular carcinoma (sHCC, diameter ≤ 3 cm) is an early malignant tumor with a relatively good prognosis. Surgical resection, liver transplantation, and radiofrequency ablation are the primary curative treatment strategies for patients with sHCC [3]. However, some patients may relapse because of microvascular invasion (MVI) after the radical operation, causing an unsatisfactory prognosis [4]. Therefore, predicting MVI of sHCC before the surgical operation could guide the clinician to choose proper strategies, then improving outcomes of patients.

Gadolinium–ethoxybenzyl–diethylenetriamine penta–acetic acid (Gd–EOB–DTPA) is well known as a perfect liver–specific contrast agent. Due to hepatic cell uptake, it can distinguish abnormal lesions from normal liver parenchyma easily and improve small lesions′ detection rate [5, 6]. Hence, gadoxetic acid is considered as the most critical contrast medium for diagnosing and detecting HCC. Besides, the studies that predicting MVI ofsHCC by Gd–EOB–DTPA enhanced magnetic resonance imaging (Gd–EOB–DTPA–MRI) before surgery were relatively immature. Some studies had attempted to predict MVI preoperatively by Gd–EOB–DTPA–MRI [7–14]. Peritumoral hyper enhancement on the arterial phase [15] and hypo–uptake on hepatobiliary phase (HBP)are known as prominent risk factors of MVI [16, 17]. However, to the best of our knowledge, few systematic study assessed the efficacy of Gd–EOB–DTPA–MRI in predicting MVI of sHCC. Our study intergrated the imaging and clinicopathological features and aimed to find effective marker sin predicting MVI of sHCC preoperatively.

## Methods

### Patients

**Fig. 1:**
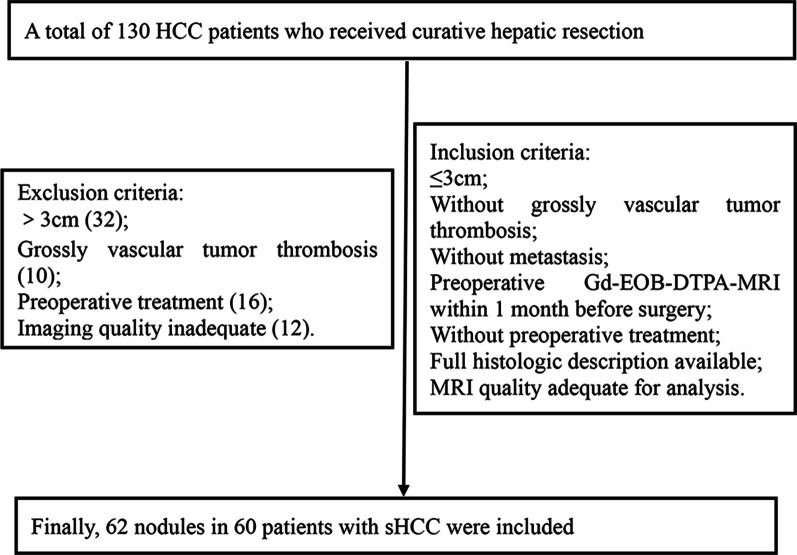
Flow chart shows study population and inclusion criteria

A total of 130 HCC patients who received curative hepatic resection at our hospital between October 2018 and October 2019 were enrolled in this study. The inclusion criteria for our study were as follow: (a) HCCs′ diameters were equal to or less than 3 cm in maximum; (b) Patients underwent preoperative Gd–EOB–DTPA–MRI within one month before surgery; (c) There were no grossly vascular tumor thrombosis or extrahepatic metastasis on preoperative imaging evaluation;(d) There were no preoperative treatments; (e) Full histologic description was available in the pathologic reports and imaging quality adequate for analysis; (f) All malignant nodules were involved for analysis in patients with multiple sHCCs. Finally, 62 nodules in 60 patients were included in the present retrospective study (Fig. 1).This study was approved by the Ethics Review Board of Harbin Medical University. The requirement of informed consent from the patients was waived because of the retrospective design of this study, and patients′ information was protected.

### MRI

#### MRI Technique

All MRI examinations were performed by a 3.0 T system (Achieva, Philips Medical Systems, Best, the Netherlands) and scanned from the top to the lower edge of the liver tissue. An axial fat–suppressed respiratory–triggered T2-weighed images (T2WI) single–shot turbo spin echo (TR/TE = 535 ms/75 ms, slice thickness/gap = 7/1 mm, FOV = 350 × 392 mm, matrix size = 232 × 199), a coronal breath–hold T2WI single–shot turbo spin echo (TR/TE = 1100 ms/80 ms, slice thickness/gap = 6/1 mm, FOV = 350 × 346 cm, matrix size = 292 × 253), an axial breath–hold dual–echo (in–phase and opposed–phase) T1–weighte dimages (T1WI) fast field–echo (TR/TE1/TE2 = 106 ms/1.15 ms/2.3 ms, slice thickness/gap = 7/1 mm, FOV = 400 × 322 cm, matrix size = 244 × 181). Dynamic MRI study was performed with a fat–suppressed three–dimensional volumetric interpolated breath–hold T1WI gradient–echo imaging. The acquisition parameters were section thickness and interval 5/2.50 mm, TR/TE = 3.60 ms/1.3–2 ms, field–of–view 320 × 427 cm, matrix size = 200 × 250. Patients were maintained at a supine position, advanced head position, and injected the Gd–EOB–DTPA contrast agent (Trade name Primovist, Bayer Schering, Germany) with a concentration of 0.25 mol/l (10 ml). The injection dose was 0.1 ml/kg body weight, and the injection flow rate was 1 ml/s, and then followed by 20 ml physiological saline. Arterial phase (AP), portal vein phase (PVP), equilibrium phase (EP), and transition period were obtainedat 25 s, 55 s, 90 s, and 180 s respectively. HBP images were obtained at 20 min after agents were injected.

### Image analysis

Preoperative MRI images were retrospectively evaluated using a Picture Archiving and Communication System (PACS; GE Medical Systems Integrated Imaging Solutions, Mt. Prospect, IL, USA) with an optimal window setting adjustment in each case. Two experienced abdominal radiologists interpreted the imaging analysis (with 12 and 20 years of experience in HCC, respectively). They were blind to clinical, pathological and MVI information.

Our study evaluated the imaging features of each sHCC and focused on the following imaging features.

#### Non–enhancement imaging

**Fig. 2:**
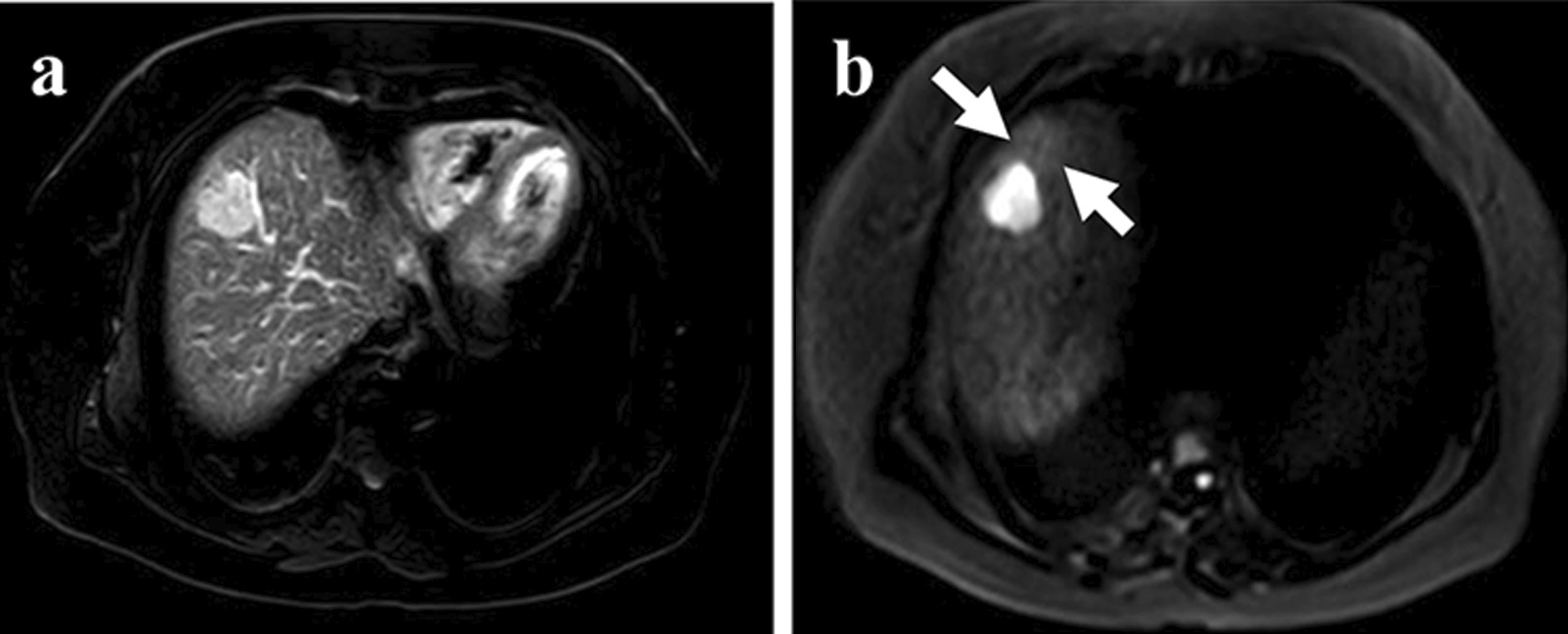
A 54-year-old woman with sHCC with MVI. **a** T2WI shows a high signal intensity hepatic mass with round shape and smooth margins. **b** DWI shows mismatch region compared to T2WI (thin arrow)

**Fig. 3:**
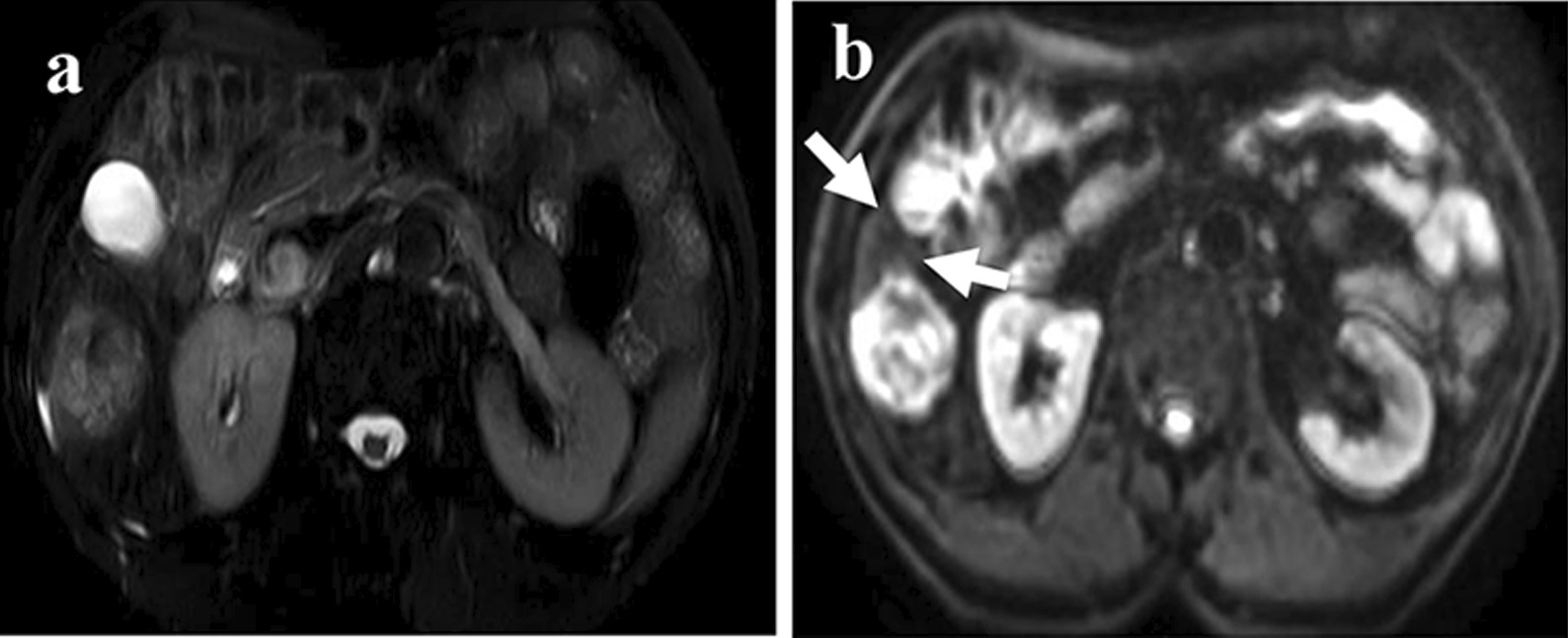
A 69-year-old woman with sHCC with MVI. **a** T2WI shows a high signal intensity hepatic mass with round shape and smooth margins. **b** DWI shows mismatch region compared to T2WI (thin arrow)

(a) Mosaic architecture: it is anancillary feature of Liver Imaging Reporting And Data Systemthat is favoring HCC in particular (American College of Radiology (ACR). Liver Reporting & Data System (LI-RADS). ACR website.www.acr.org/Clinical-Resources/Reporting-and-Data-Systems/LI-RADS.). Mosaic architecture refers to the difference present within mass of randomly distributed internal nodules or compartments differing in enhancement, attenuation, intensity, shape, and size and often separated by fibrous separations [18]. It is characteristic of a heterogeneous signal on T2WI [19]; (b) Intralesional fat: the chemical shift of intralesional fat cause an area shows significantly lower signal intensity (SI) on opposed–phase imagesT1WI compared with the SI in-phase images [20], which confirmed the presence of steatosis [21]; (c) intratumor hemorrhage: with ahyper–SI on unenhanced T1WI and a hypo–SI on T2WI [21]; (d) Iso/Hyperintense SI (T1WI):isointense (hyper–isointense) SI in the lesion on T1WI; (e) T2–weighted and diffusion-weighted imaging (T2–DW) mismatch: T2–DW mismatch mainly means a morphological mismatch, with a larger mass on thediffusion weighted imaging (DWI) images than T2WI images. Because of the image distortion on the DWI images, it was not accurate that only compare the lesion diameter or area on DWI with T2WI. So, we added the other requirement. The mismatch region's intensity on DWI was lower than the tumor itself but higher than the liver parenchyma. These conditions are the same as the previous study [22] (Figs. 2, 3); (f) Morphology: it was assessed on T2WI and categorized as round, lobulated and irregular shape.

#### Enhancement without HBP imaging

(g) AP hyper–enhancement: it was referred to AP enhancement that unequivocally greater than the background of hepatic parenchyma rather than a rim structure; (h) Washout: it was based on AP hyper–enhancement and followed by a lower SI than the normal hepatic parenchyma on PVP and/or EP [23]; (i) Rim enhancement: it was defined as irregular ring–like enhancement with relatively hypovascular central areas in the AP; (j) Capsule enhancement: it was assessed during EP and defined as thin, linear and enhanced structure surrounding the tumor; (k) AP halo sign: it was described as an irregular and ring–like enhancement sign that adjacent to tumor border.

#### HBP imaging

**Fig. 4:**
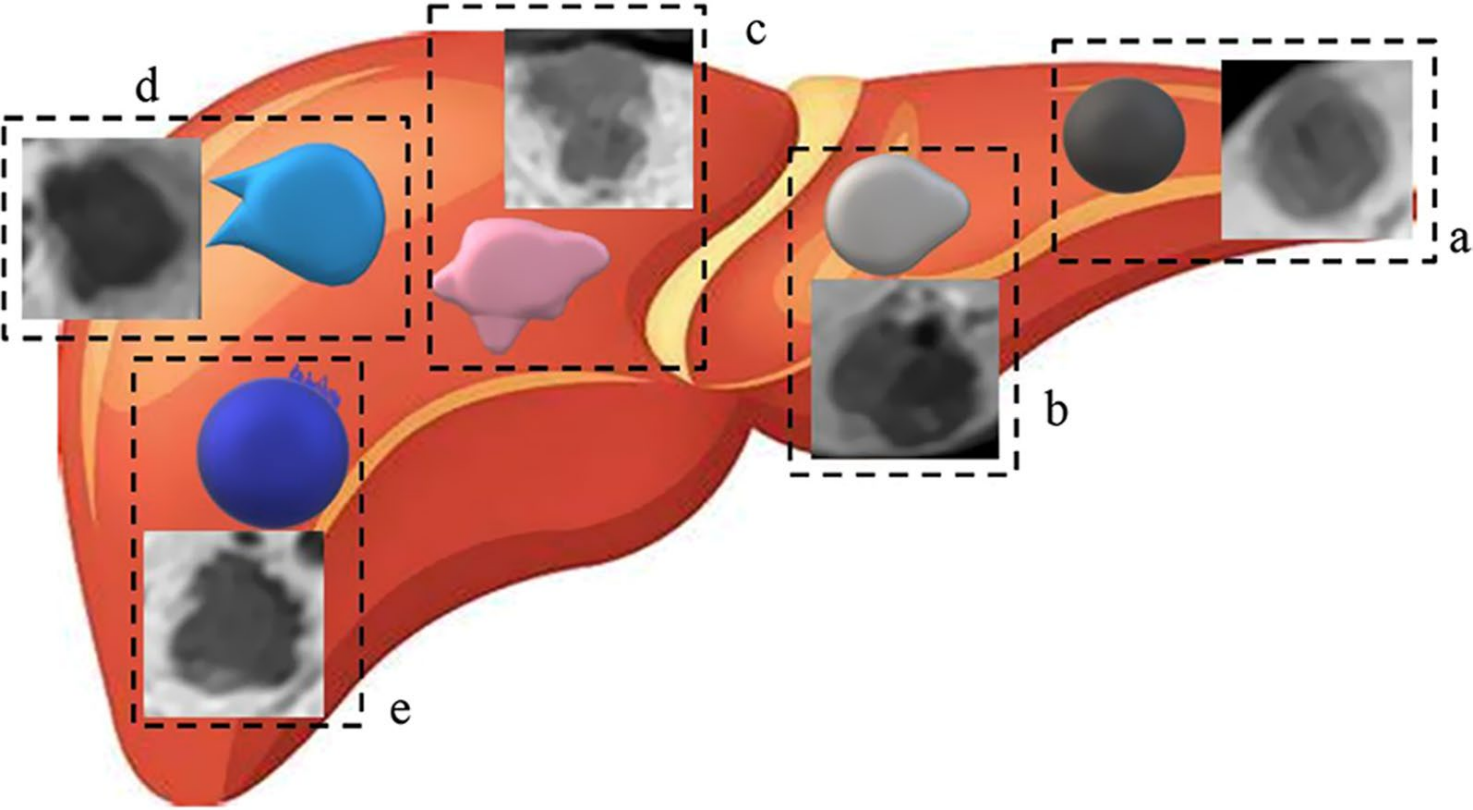
Illustration of five morphologic features on hepatobiliary phase and sketch map. **a** round shape of sHCC with 6.8% (3/44) MVI–positive. **b** Lobulated shape of sHCC with 45.8% (11/24) MVI–positive. **c** Irregular shape of sHCC with 80% (4/5) MVI–positive. **d** Spicule sign of sHCC with 80% (12/15) MVI–positive. **e** bush sign of sHCC with 66.7% (8/12) MVI–positive

(l) HBP high uptake: it shows an increased tumor uptake of contrast agents and as lightly high SI in the lesion area [24, 25]. (m) HBP halo sign: it was a hypo–intensity ring around tumors [15]. (n) HBP morphology: it was categorized on T2WI images. (o) HBP spicule sign: it was a thick, blurred, and pseudopodal structure. (p) HBP brush sign: it was the edges of the tumor and appeared as a blurry, small corner projection (Fig. 4).

### Pathology

The diagnosis of MVI was based on histologic specimens obtained from surgical resection in all patients. Based on the World Health Organization classification system, an experienced pathologist (with nine years of experience) who was blind to all clinical and MRI results confirmed the histologic diagnosis and assessed histologic grade, vascular invasion, and tumor diameter.

According to the classification proposed by the General Rules of the Clinical and Pathological Study of Primary Liver Cancer in Japan [26], we classified the tumor grade into highly, moderately and lowly differentiated. Besides, the predominant grade was assigned when grades coexisted. The tumor diameter was defined as the maximum diameter of the resected tumor specimen.

We consider that pathological materials should combine with the MRI findings. Once patients with MVI areassessed before surgery, the scope of surgery or pathological materials should be expanded in the area where the MVI is suspected.

### Statistical analysis

We used SPSS software (version 22.0, IBM) to analyze all data. Categorical variables were compared by the chi–square test and followed by a multivariate logistic regression analysis when the variables were significant in the chi–square test. Then we divided all features into Non–HBP (non–enhancement and enhancement without HBP), HBP and the combination of those two phases. We performed the logistic regression analysis to assess potential imaging predictors for MVI in different phases. Variables with *p* < 0.05 in the uni variate analysis were applied to multivariate logistic regression analysis. Features with p value less than 0.05 were considered statistically significant. A receiver operating characteristic (ROC) curves were performed to evaluate the diagnostic ability of each phase and their combination. The value of sensitivity (SE), specificity (SP), accuracy, positive predictive value (PPV), and negative predictive value (NPV) in these three parts were also calculated. Additionally, to test the generalizability of the tested and the inter observer variability of the radiological features, we assessed the intra class correlation coefficient test (ICC) (ĸ = 0.00–0.20, poor agreement; ĸ = 0.21–0.40, fair agreement; ĸ = 0.41–0.60, moderate agreement; ĸ = 0.61–0.80, good agreement; ĸ = 0.81–1.00, excellent agreement) [27].

## Results

### Clinicopathological features results

Among 62 sHCC nodules (60 patients), 19 nodules (19 patients) had MVI, while 43 nodules (41 patients) had no MVI. The study included 47 men and 13 women, with a median age of 55 (34–83). Comparisons of clinical characteristics between sHCC with and without MVI were summarized in Table 1. Alanine aminotransferase (ALT) was higher in sHCC patients with MVI than those without MVI. Concerning histologic features, sHCC with MVI showed worse tumor differentiation than those without MVI (*p* < 0.05).

**Table 1:** The clinical and pathological features of the patients

Features	MVI-(n = 41/43)	MVI + (n = 19/19)	*p* Value
Sex			0.276
Man	30 (73.2%)	17 (89.5%)	
Woman	11 (26.8%)	2 (10.5%)	
Age (year)			0.116
< 52	30 (50%%)	10 (16.7%)	
3 52	11 (18.3%)	9 (15%)	
History of drinking			0.813
Yes	12 (29.3%)	5 (26.3%)	
No	29 (70.7%)	14 (73.7%)	
History of smoking			0.985
Yes	15 (36.6%)	7 (36.8%)	
No	26 (63.4%)	12 (63.2%)	
Cirrhosis			0.287
Yes	32 (78.0%)	17 (89.5%)	
No	9 (22.0%)	2 (10.5%)	
CEA (ng/ml)			0.908
> 5	2 (3.6%)	1 (1.7%)	
< 5	37 (66.1%)	16 (28.6%)	
Ca199 (u/ml)			0.528
> 40	6 (10.5%)	4 (7%)	
= S40	33 (57.9%)	14 (24.6%)	
AFP (ng/ml)			0.094
> 25	15 (25.9%)	11 (19.0%)	
	25 (43.1%)	7 (12.1%)	
ALT (u/l)			**0.003**
> 40	5 (8.3%)	9 (15%)	
< 40	36 (60%)	10 (16.6%)	
AST (u/l)			
> 35	12 (20%)	9 (15%)	0.172
< 35	29 (48.3%)	10 (16.7%)	
GGT (u/l)			0.052
> 40	17 (28.3%)	13 (21.7%)	
< 40	24 (40%)	6 (10%)	
Albumin (g/l)			1.000
< 40	29 (48.3%)	13 (21.7%)	
> 40; < 55	12 (20%)	6 (10%)	
> 55	0 (0%)	0 (0%)	
HBV			0.061
Yes	22 (53.7%)	15 (78.9%)	
No	19 (46.3%)	4 (21.1%)	
Pathology classification			0.003
Low	1 (2.3%)	6 (31.6%)	
Medium	23 (53.5%)	8 (42.1%)	
High	19 (44.2%)	5 (26.3%)	
Tumor size (cm)			0.065
> 2.75	19 (31.7%)	10 (16.6%)	
< 2.75	22 (36.7%)	9 (15%)	

Except where indicated otherwise, data are number (%) of patients. Categoric imaging variables were analyzed by the chi-square test. CEA, carcinoembryonicantigen; CA199, carbohydrate antigen 199; AFP, alpha-fetoprotein; AST, aspartate aminotransferase; ALT, alanine aminotransferase; GGT, gamm glutamyl transferase; HBV, hepatitis B virus

### Imaging features results

#### Univariate analysis of clinical, pathological and imaging features

Among MRI features showed in Table 2, on non–enhancement phase, T2–DW mismatch was significantly more frequent in sHCC with MVI than those without MVI (*p* < 0.05). On enhancement phase without HBP, rim enhancement, capsule enhancement and AP halo sign were associated with MVI (*p* < 0.05). All imaging features including high uptake, halo sign, spicule sign and brush sign on HBP were more common in sHCC with MVI than those without MVI (*p* < 0.05). Tumor morphology of lobulated and irregular shape in sHCC with MVI was significantly more frequent than that of sHCC without MVI (*p* < 0.05).Besides, the round shape showed more common in sHCC without MVI than those with MVI (*p* < 0.05). Themorphology features of sHCC are presented in Fig. 4. A round shape of sHCC with MVI accounts for6.80% (3/44),a lobulated shape accounts for 45.80%(11/24), an irregular shape accounts for 80.00%(4/5), a spicule sign accounts for 80.00%(12/15) and a brush sign accounts for 66.70%(8/12), respectively.

**Table 2:** The imaging features of the patients

Features	MVI-(n = 43)	MVI + (n = 19)	*p* Value
*Non-enhancement*			
Mosaic architecture			
Yes	7 (16.3%)	1 (5.3%)	0.233
Intralesional fat			
Yes	16 (37.2%)	4 (21.1%)	0.210
Intratumor hemorrhage			
Yes	2 (4.7%)	3 (15.8%)	0.138
Iso/hyper-intense SI (T1WI)			
Yes	13 (30.2%)	3 (15.8%)	0.231
T2-DW mismatch			
Yes	1 (2.3%)	3 (15.8%)	0.047
Morphology (T2WI)			
Round shape	20 (46.5%)	10 (52.6%)	0.250
Lobulated	20 (46.5%)	6 (31.6%)	
Irregular shape	3 (7.0%)	3 (15.8%)	
*Enhancement without HBP*			
AP hyper-enhancement			
Yes	32 (74.4%)	14 (73.7%)	0.951
Washout			
No enhancement	4 (9.3%)	1 (5.3%)	0.804
Enhance and without Washout	7 (16.3%)	4 (21.1%)	
Enhance and washout	32 (74.4%)	14 (73.7))	
Rim enhancement			
Yes	5 (11.6%)	9 (47.4%)	0.006
Capsule enhancement			
Enhance and complete	16 (37.2%)	5 (26.3%)	< 0.001
Enhance and Uncomplete	23 (53.5%)	2 (10.5%)	
No enhance	4 (9.3%)	12 (63.2%)	
AP halo signs			
Yes	5 (11.6%)	9 (47.4%)	0.006
HBP			
HBP high uptake			
Yes	17 (39.5%)	2 (10.5%)	0.022
HBP halo sign			
Yes	4 (9.3%)	8 (42.1%)	0.013
HBP morphology			
Round shape	29 (67.4%)	4 (21.1%)	< 0.001
Lobulated	11 (25.6%)	5 (26.3%)	
Irregular shape	3 (7.0%)	10 (52.7%)	
HBP spicule sign			
Yes	3 (7.0%)	12 (63.2%)	< 0.001
HBP brush sign			
Yes	4 (9.3%)	8 (42.1%)	0.003

Except where indicated otherwise, data are number (%) of patients. Categoric imaging variables were analyzed by the chi-square test. SI, signal intensity; T2-DW, T2-weighted and diffusion-weighted imaging; T2WI, T2-weighted; AP, arterial phase; HBP, hepatobiliary phase

### Univariate and multivariate logistical regression analyses of clinical, pathological and imaging features

In univariate logistical regression analysis, ALT, rim enhancement and spicule sign were associated with MVI (*p* < 0.05). And the association remained significant for rim enhancement and spicule sign in multivariate logistical regression analysis (*p* < 0.05), which were summarized in Table3.

**Table 3:** Univariate and multivariate analysis results

Features	Univariate analysis		Multivariate analysis	
	OR	*p* Value	OR	*p* Value
ALT	2.406	**0.155**		
Without HBP				
T2-DW mismatch	5.857	0.384		
Capsule enhancement	1.239	0.794		
Rim enhancement	12.743	**0.021**	10.783	**0.011**
AP halo sign	0.405	0.456		
HBP alone				
HBP high uptake	0.083	0.071		
HBP halo sign	0.030	0.197		
HBP morphology	2.488	0.077		
HBP spicule sign	78.469	**0.023**	31.653	**0.000**
HBP brush sign	2.488	0.779		

Multivariate analysis was performed with univariate *p* value < 0.05. ALT, alanine aminotransferase; HBP, hepatobiliary phase; AP: arterial phase; T2-DW: T2-weighted and diffusion-weighted imaging

Inter observer agreement for capsule enhancement (κ = 0.844), rim enhancement (κ = 0.749) and HBP spicule sign (κ = 0.876) was good or excellent (Table 4).

**Table 4:** The interobserver variability for radiological features

	Kappa	CI 95%
*Non-enhancement*		
Mosaic architecture	0.763	0.545–0.981
Intralesional fat	0.893	0.777–1.000
Intratumor hemorrhage	0.816	0.569–1.000
Iso/Hyper-intense SI (T1WI)	0.876	0.740–1.000
T2-DW mismatch	0.703	0.390–1.000
Morphology (T2WI)	0.871	0.775–0.966
*Enhancement without HBP*		
AP hyper-enhancement	0.876	0.741–1.000
Washout	0.800	0.657–0.943
Rim enhancement	0.749	0.561–0.936
Capsule enhancement	0.844	0.718–0.970
AP halo sign	0.760	0.582–0.937
*HBP*		
HBP high uptake	0.926	0.826–1.000
HBP halo sign	0.857	0.664–1.000
HBP morphology	0.951	0.894–1.000
HBP spicule sign	0.876	0.741–1.000
HBP brush sign	0.858	0.704–1.000

k = 0.00–0.20, poor agreement; k = 0.21–0.40, fair agreement; **k** = 0.41–0.60, moderate agreement; **k** = 0.61–0.80, good agreement; **k** = 0.81–1.00, excellent agreement. T1WI, T1-weighted; AP, arterial phase; T2-DW: T2-weighted and diffusion-weighted imaging; T2WI, T2-weighted; HBP, hepatobiliary phase

### Evaluate the diagnostic ability of gadoxetic acid-enhanced MRI

**Fig. 5.**
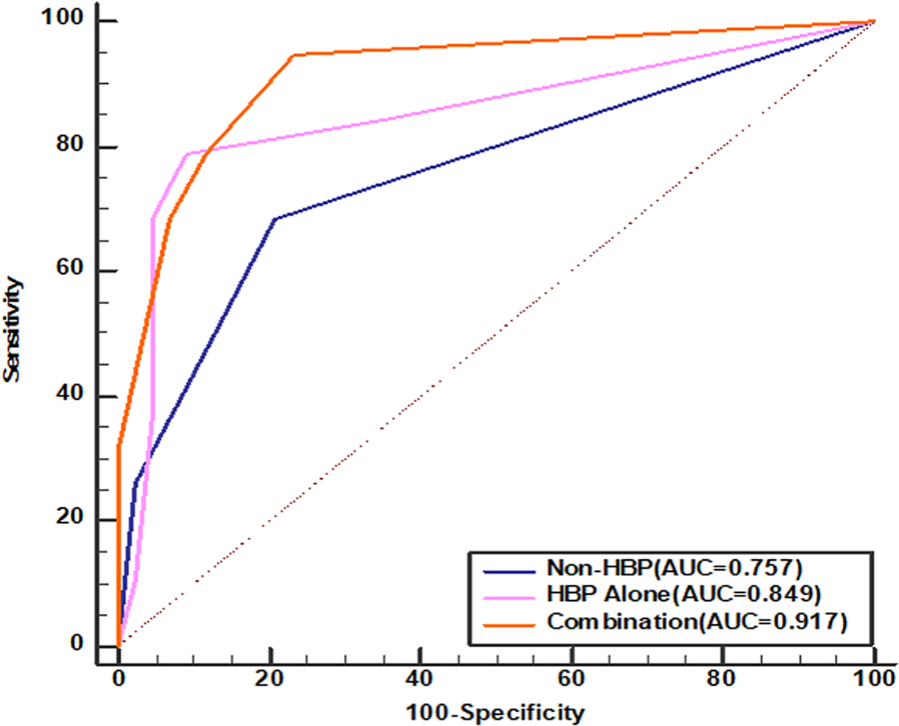
Comparison of receiver operating characteristics (ROC) curves for prediction of microvascular invasion

We analyzed the diagnostic efficacy of two significant imaging phases and their combination for predicting MVI (Fig. 5, Table 5).When two of these imaging phases were combined, the area under the ROC curve (AUROC) was higher than the non–HBP phase or HBP separately.

**Table 5:** Diagnostic efficacy of three image sequences for MVI in sHCC

	SE (%)	SP (%)	Accuracy (%)	PPV (%)	NPV (%)	AUC
Non-HBP	68.42	79.07	75.81	97.67	26.32	0.757
HBP	78.95	90.70	87.10	93.02	73.68	0.849
Combination	94.74	76.74	85.48	88.37	78.95	0.917

SE, sensitivity; SP, specificity; PPV, positive predictive value; NPV, negative predictive value; AUROC, area under curve; HBP, hepatobiliary phase

The analysis of SE, SP, accuracy, PPV, and NPV were presented in Table 5.Compared to non–HBP phase or HBP, the combination of these two imaging phases showed significantly higher SE, accuracy and NPV. Consequently, the combination of these two imaging phases can predict MVI more accurately compared with them separately.

## Discussion

The purpose of this research was to predict MVI in sHCC by imaging features, clinical features and pathological features, providing a direction for clinicians to choose a superior treatment strategy then improving patients′ survival.

Our research used a special contrast agent and achieved satisfactory results. Gd–EOB–DTPA, a hepatocyte–specific contrast agent, has dual enhanced information which not only exhibits a multi–phase dynamic enhancement similar to extra cell contrast medium by reducing the T1 relaxation time of tissues but also can acquire high uptake images of normal liver parenchyma after administrating the agent for 20 min. Therefore, this particular agent can deliver more information about the HCC from AP, PVP, EP and HBP. It is more effective than traditional MRI in diagnosing early HCC and sHCC [28], especially in the detection of tiny lesions [29]. Meanwhile, because of the background of high uptake of normal liver tissue on HBP, the morphology of the tumor appears much clearly [30].

Gd–EOB–DTPA contrast agent has many advantages in hepatic MRI examination, however, transient respiratory motion artifacts result in a diagnostic limitations about the observation of AP information. Finding more significant features to improve MVI diagnosis by observing the morphology of the lesions during the HBP is extremely necessary. Up to date, studies discuss the features that associated with the occurrence of MVI in sHCC were rare. Xu etal [31] proved that the high ADC value and irregular circumferential enhancement were independent predictors of MVI, which was consistent with our research results. Kim and his colleagues showed that all sHCCs with MVI were characterized by a typical dynamic pattern, hyper intensity on T2WI, DWI, atypical dynamic pattern. Instead, the size of diameter was less than 1 cm indicated the absence of MVI [32]. Our research also analyzed the signal intensity on AP and showed an insignificant result (MVI–negative32/43, 74.40% vs. MVI–positive 14/19, 73.70%,p > 0.05). The difference between Kim′s and ours may be caused by the specific classification of tumor size and the small sample size. Considering the number of samples in our research, the analysis of ADC values may produce deviations in results, so our study did not conduct further investigation. However, the relationship between the ADC value and MVI is worthfurther discussion. Besides, Ryu et al. declared including the clinical characteristics that tumor diameter ≥ 2 cm, AFP ≥ 200 ng/ml and gamma-glutamyl transferase ≥ 40 u/l can predict MVI in sHCC [14]. However, our research did not get the same results, and we regarded a relatively higher ALT value as a significant feature in MVI prediction (*p* < 0.05).

In our study, by comparing clinic pathological features and imaging features of non–HBP and HBP, we found that the HBP features provided us more insights for MVI diagnosis (Table 2). All of the characteristics we discovered were significantly different between MVI–positive and MVI–negative group. About 3/5 of characteristics (HBP tumor morphology, spicule sign and brush sign) were morphology features. Up to date, several studies proved that the irregular tumor shape was associated with the presence of MVI [33, 34]. In particular, our research is the first attempt to classify the irregular shape of sHCC in detail. As a result, we should pay attention to the spicule sign that is contained in most MVI–positive patients (12/19, 63.20%), and few (3/43, 7.00%) in MVI–negative sHCCs. Sign of spicule between the lobe and burr represents tumor infiltration into the base of the lobes. As an independent risk factor, our research showed a better performance in diagnosing MVI than irregular shape and brush features. The spicule sign was quoted from lung cancer and it′s detection rate was approximately 90.00%, which was a central differential diagnostic marker of pulmonary nodules [35] and predicted poor biological behavior [36]. According to our cases, it is also appropriate for the judgment of the biological behavior of sHCC. Besides, it should be noted that the morphological change is more easily discovered and acceptable for the clinician than other complex enhancement and signal features.

Except for spicule sign on HBP images, rim enhancement was another independent risk factor for diagnosing MVI on non–HBP images. There were 9 cases with MVI in all cases of rim enhancement (9/13, 69.23%), and 12 cases with MVI in all cases of none capsule enhancement (12/16, 75.00%). Rim enhancement on AP reflected the internal tissue of fiber necrosis, liquefaction or calcification caused by the insufficient blood supply. We speculated obstruction of surrounding from MVI was likely to further exacerbate the blood loss, intensifying internal necrosis as well as margin enhancement.

Finally, we divided all features into two phases, non–HBP (non–enhancement and enhancement without HBP)and HBP alone. And we compared the diagnostic capability of each phase and the combination phase. The combining phase group achieved the highest AUROC (0.917) than the two phases alone. Meanwhile, HBP image features give the best result inaccuracy and SP, which was entirely credible in predicting MVI in sHCC. To the best of our knowledge, the main reasons that the SE of the combined phase images and SP of HBP images (> 90%) in our research might be explained as follows. Firstly, Gd–EOB–DTPA–MRI can accurately discriminate cancer boundaries. Moreover, the distinction in signal between lesion tissues and surrounding normal liver parenchyma is more apparent in the HBP of Gd–EOB–DTPA–MRI than conventional contrast agents [37, 38], which contributes to the highest SP in HBP than non–HBP. Secondly, owing to the high performance of radiomics in the stable calculation, repeatability, indefatigability, and no interference of human subjectivity. Our research made a much higher SE and SP than those ever reported [39–42]. Thirdly, we extracted almost the whole sHCC features on HBP, including the three–dimensional features (e.g., morphology and smoothness), making the study much more credible and representative. Moreover, we found that the NPV of non–HBP images was very low, which suggested a high possibility of missed diagnosis. Thus, we suggested that the characteristics of HBP images alone as predominant indicators, especially morphologic features, which could increase radiologist confidence in diagnosing sHCC with MVI.

However, our study had the following limitations. Firstly, this was a retrospective study that may have included selection bias. Secondly, the number of the sample was insufficient, which may produce errors in data analysis. Thirdly, it was a single center study, which may also cause sample selection bias. Therefore, it is worthwhile to conduct further research to verify our results.

## Conclusions

This study suggested that Gd–EOB–DTPA–MRI was recommended as a routine preoperative examination for sHCC to improve the accuracy of MVI diagnosis. The morphologies of HBP imaging, especially sign of spicules, showed high accuracy in diagnosing MVI of sHCC.

## Data Availability

The datasets generated and/or analyzed in the current study are not publicly available due to patient privacy protection but are available from the corresponding author on reasonable request.

## Abbreviations

HCC: hepatocellular carcinoma
sHCC: small hepatocellular carcinoma
MVI: microvascular invasion
Gd–EOB–DTPA–MRI: gadolinium–ethoxybenzyl–diethylenetriamine penta–acetic acid enhanced magnetic resonance imaging
T2WI: T2–weighted
T1WI: T1–weighted images
AP: arterial phase
PVP: portal vein phase
EP: equilibrium phase
MRI: magnetic resonance imaging
PACS: picture archiving and communication system
SI: signal intensity
T2–DW: T2–weighted and diffusion–weighted imaging
ROC: receiver operating characteristic
SE: sensitivity
SP: specificity
PPV: positive predictive value
NPV: negative predictive value
HBV: hepatitis B virus
AST: aspartate aminotransferase
GGT: gamma–glutamyl transferase
ALT: alanine aminotransferase
OR: odds ratio
CI: confidence interval
AUROC: the area under the ROC curve
CEA: carcinoembryonic antigen
CA199: carbohydrate antigen 199
AFP: alpha–fetoprotein

## References

[1] Wu J, Yang S, Xu K, Ding C, Zhou Y, Fu X (2018). Patterns and trends of liver cancer incidence rates in Eastern and Southeastern Asian countries (1983–2007) and predictions to 2030. Gastroenterology.

[2] Villanueva A (2019). Hepatocellular carcinoma. N Engl J Med.

[3] Kanwal F, Befeler A, Chari RS, Marrero J, Kahn J, Afdhal N (2012). Potentially curative treatment in patients with hepatocellular cancer–results from the liver cancer research network. Aliment Pharmacol Ther.

[4] Fukuda S, Itamoto T, Nakahara H, Kohashi T, Ohdan H, Hino H (2005). Clinicopathologic features and prognostic factors of resected solitary small-sized hepatocellular carcinoma. Hepato Gastroenterol..

[5] Golfieri R, Renzulli M, Lucidi V, Corcioni B, Trevisani F, Bolondi L (2011). Contribution of the hepatobiliary phase of Gd-EOB-DTPA-enhanced MRI to dynamic MRI in the detection of hypovascular small (</= 2 cm) HCC in cirrhosis. Eur Radiol.

[6] Lee YJ, Lee JM, Lee JS, Lee HY, Park BH, Kim YH (2015). Hepatocellular carcinoma: diagnostic performance of multidetector CT and MR imaging-a systematic review and meta-analysis. Radiology.

[7] Lee S, Kim SH, Lee JE, Sinn DH, Park CK (2017). Preoperative gadoxetic acid-enhanced MRI for predicting microvascular invasion in patients with single hepatocellular carcinoma. J Hepatol.

[8] Zhu F, Yang F, Li J, Chen W, Yang W (2019). Incomplete tumor capsule on preoperative imaging reveals microvascular invasion in hepatocellular carcinoma: a systematic review and meta-analysis. Abdom Radiol (New York).

[9] Zhao W, Liu W, Liu H, Yi X, Hou J, Pei Y (2018). Preoperative prediction of microvascular invasion of hepatocellular carcinoma with IVIM diffusion-weighted MR imaging and Gd-EOB-DTPA-enhanced MR imaging. PLoS ONE.

[10] Huang M, Liao B, Xu P, Cai H, Huang K, Dong Z (2018). Prediction of microvascular invasion in hepatocellular carcinoma: preoperative Gd-EOB-DTPA-dynamic enhanced MRI and histopathological correlation. Contrast Media Mol Imaging.

[11] Wu TH, Hatano E, Yamanaka K, Seo S, Taura K, Yasuchika K (2016). A non-smooth tumor margin on preoperative imaging predicts microvascular invasion of hepatocellular carcinoma. Surg Today.

[12] Zeng F, Chen B, Zeng J, Wang Z, Xiao L, Deng G (2019). Preoperative neutrophil-lymphocyte ratio predicts the risk of microvascular invasion in hepatocellular carcinoma: A meta-analysis. Int J Biol Markers.

[13] Chen J, Zhou J, Kuang S, Zhang Y, Xie S, He B (2019). Liver imaging reporting and data system category 5: MRI predictors of microvascular invasion and recurrence after hepatectomy for hepatocellular carcinoma. AJR Am J Roentgenol.

[14] Ryu T, Takami Y, Wada Y, Tateishi M, Hara T, Yoshitomi M (2019). A clinical scoring system for predicting microvascular invasion in patients with hepatocellular carcinoma within the milan criteria. J Gastrointest Surg.

[15] Reginelli A, Vanzulli A, Sgrazzutti C, Caschera L, Serra N, Raucci A (2017). Vascular microinvasion from hepatocellular carcinoma: CT findings and pathologic correlation for the best therapeutic strategies. Med Oncol.

[16] Lee S, Kim SH, Lee JE, Sinn DH, Park CK (2017). Preoperative gadoxetic acid-enhanced MRI for predicting microvascular invasion in patients with single hepatocellular carcinoma. J Hepatol..

[17] Wei Y, Huang Z, Tang H, Deng L, Yuan Y, Li J, Song B (2019). IVIM improves preoperative assessment of microvascular invasion in HCC. Eur Radiol..

[18] Piscaglia F, Wilson SR, Lyshchik A, Cosgrove D, Dietrich CF, Jang HJ (2017). American college of radiology contrast enhanced ultrasound liver imaging reporting and data system (CEUS LI-RADS) for the diagnosis of hepatocellular carcinoma: a pictorial essay. Ultraschall in der Medizin..

[19] Honda H, Ochiai K, Adachi E, Yasumori K, Hayashi T, Kawashima A (1993). Hepatocellular carcinoma: correlation of CT, angiographic, and histopathologic findings. Radiology.

[20] Horvat N, Monti S, Oliveira BC, Rocha CCT, Giancipoli RG, Mannelli L (2018). State of the art in magnetic resonance imaging of hepatocellular carcinoma. Radiol Oncol.

[21] Kim SS, Kim SH, Song KD, Choi SY, Heo NH (2020). Value of gadoxetic acid-enhanced MRI and diffusion-weighted imaging in the differentiation of hypervascular hyperplastic nodule from small (<3 cm) hypervascular hepatocellular carcinoma in patients with alcoholic liver cirrhosis: a retrospective case-control study. J Mag Resonan Imaging JMRI.

[22] Yang C, Wang H, Sheng R, Ji Y, Rao S, Zeng MJCI (2017). Microvascular invasion in hepatocellular carcinoma: is it predictable with a new, preoperative application of diffusion-weighted imaging?. Clin Imaging.

[23] Yoon JH, Park JW, Lee JM (2016). Noninvasive diagnosis of hepatocellular carcinoma: elaboration on Korean liver cancer study group-national cancer center Korea practice guidelines compared with other guidelines and remaining issues. Korean J Radiol.

[24] Jw C, Jm L, Sj K, Jh Y, Jh B, Jk H (2013). Hepatocellular carcinoma: imaging patterns on gadoxetic acid-enhanced MR Images and their value as an imaging biomarker. Radiology.

[25] Kitao A, Zen Y, Matsui O, Gabata T, Kobayashi S, Koda W (2010). Hepatocellular carcinoma: signal intensity at gadoxetic acid-enhanced MR Imaging–correlation with molecular transporters and histopathologic features. Radiology..

[26] Kudo M, Kitano M, Sakurai T, Nishida N (2015). General rules for the clinical and pathological study of primary liver cancer, nationwide follow-up survey and clinical practice guidelines: the outstanding achievements of the liver cancer study group of Japan. Digest Dis (Basel Swit).

[27] Shrout PE, Fleiss JL (1979). Intraclass correlations: uses in assessing rater reliability. Psychol Bull.

[28] Li X, Wang X, Zhao D, Sun J, Liu J, Lin D (2020). Application of Gd-EOB-DTPA-enhanced magnetic resonance imaging (MRI) in hepatocellular carcinoma. World J Surg Oncol.

[29] Semaan S, Vietti Violi N, Lewis S, Chatterji M, Song C, Besa C (2020). Hepatocellular carcinoma detection in liver cirrhosis: diagnostic performance of contrast-enhanced CT vs. MRI with extracellular contrast vs. gadoxetic acid. Eur Radiol.

[30] Hu H, Zheng Q, Huang Y, Huang XW, Lai ZC, Liu J (2017). A non-smooth tumor margin on preoperative imaging assesses microvascular invasion of hepatocellular carcinoma: a systematic review and meta-analysis. Sci Rep..

[31] Xu P, Zeng M, Liu K, Shan Y, Xu C, Lin J (2014). Microvascular invasion in small hepatocellular carcinoma: is it predictable with preoperative diffusion-weighted imaging?. J Gastroenterol Hepatol.

[32] Kim MJ, Lee M, Choi JY, Park YN (2012). Imaging features of small hepatocellular carcinomas with microvascular invasion on gadoxetic acid-enhanced MR imaging. Eur J Radiol.

[33] Huang M, Liao B, Xu P, Cai H, Huang K, Dong Z, Feng ST. et al. Prediction of microvascular invasion in hepatocellular carcinoma: preoperative Gd-EOB-DTPA-dynamic enhanced MRI and histopathological correlation. Contrast Med Mol Imaging. 2018;2018:9674565. 10.1155/2018/9674565 PMC5828041 29606926

[34] Kim H, Park MS, Choi JY, Park YN, Kim MJ, Kim KS (2009). Can microvessel invasion of hepatocellular carcinoma be predicted by pre-operative MRI?. Eur Radiol..

[35] Zhang X, Yan HH, Lin JT, Wu ZH, Liu J, Cao XW (2014). Comparison of three mathematical prediction models in patients with a solitary pulmonary nodule. Chin J Cancer Res.

[36] Shi Z, Wang Y, He X (2016). Differential diagnosis of solitary pulmonary nodules with dual-source spiral computed tomography. Exp Therapeut Med.

[37] Joo I, Lee JM (2016). Recent advances in the imaging diagnosis of hepatocellular carcinoma: value of gadoxetic acid-enhanced MRI. Liver Cancer.

[38] Choi JW, Lee JM, Kim SJ, Yoon JH, Baek JH, Han JK (2013). Hepatocellular carcinoma: imaging patterns on gadoxetic acid-enhanced MR Images and their value as an imaging biomarker. Radiology.

[39] Feng ST, Jia Y, Liao B, Huang B, Zhou Q, Li X (2019). Preoperative prediction of microvascular invasion in hepatocellular cancer: a radiomics model using Gd-EOB-DTPA-enhanced MRI. Eur Radiol.

[40] Cha DI, Jang KM, Kim SH, Kim YK, Kim H, Ahn SH (2020). Preoperative prediction for early recurrence can be as accurate as postoperative assessment in single hepatocellular carcinoma patients. Korean J Radiol.

[41] Kim KA, Kim MJ, Jeon HM, Kim KS, Choi JS, Ahn SH (2012). Prediction of microvascular invasion of hepatocellular carcinoma: usefulness of peritumoral hypointensity seen on gadoxetate disodium-enhanced hepatobiliary phase images. J Magnet Resonan Imaging JMRI.

[42] Ahn SY, Lee JM, Joo I, Lee ES, Lee SJ, Cheon GJ (2015). Prediction of microvascular invasion of hepatocellular carcinoma using gadoxetic acid-enhanced MR and (18)F-FDG PET/CT. Abdom Imaging.

